# Supplementary material for: Ethyl pyruvate protects SHSY5Y cells against 6-hydroxydopamine-induced neurotoxicity by upregulating autophagy
Source: PLoS One. 2023 Feb 16;18(2):e0281957. doi: 10.1371/journal.pone.0281957 (PMC9934379; doi:10.1371/journal.pone.0281957)
Supplement: S1 Raw data — (ZIP) [file pone.0281957.s001.zip › PLOS_One-Minimal data setF.pdf]

Fig. #	Mean	S.D	S.E	Statistical method used	P value	# samples
Fig1A						
0	100.00	1.97	0.99	One way ANOVA post-hoc test	*P <0.05	4
1	106.05	14.58	7.29			4
2.5	112.08	8.15	4.08			4
5	100.01	16.57	8.28			4
10	43.37	20.63	10.32			4
Fig1B						
Ct	100.00	2.48	1.24	One way ANOVA post-hoc test	**P <0.01 ## P <0.01 VS 6OHDA	4
6OHDA	30.07	4.48	2.24			4
EP1	48.62	6.23	3.12			4
EP2.5	68.90	3.47	1.73			4
EP5	68.31	2.74	1.37			4
Fig2A						
Ct	1.00	0.12	0.07	One way ANOVA post-hoc test	*P <0.05 #P <0.05 VS 6OHDA	3
6OHDA	4.02	0.49	0.28			3
EP1	2.91	0.73	0.42			3
EP2.5	2.49	0.63	0.37			3
EP5	1.45	0.18	0.11			3
Fig2B						
Ct	1.00	0.05	0.03	One way ANOVA post-hoc test	**P <0.01 #P <0.05 VS 6OHDA	3
6OHDA	17.11	0.41	0.24			3
EP1	10.92	4.37	2.52			3
EP2.5	7.64	3.75	2.17			3
EP5	4.46	2.28	1.32			3
Fig3A						
Ct	1.00	0.07	0.04	One way ANOVA post-hoc test	*P <0.05 #P <0.05 VS 6OHDA	3
6OHDA	2.21	0.49	0.28			3
EP1	1.15	0.09	0.05			3
EP2.5	0.85	0.38	0.22			3
EP5	0.92	0.46	0.27			3
Fig3C						
Ct	1.00	0.05	0.03	One way ANOVA post-hoc test	**P <0.01 #P <0.05VS 6OHDA ##P <0.01 VS 6OHDA	3
6OHDA	2.85	0.10	0.06			3
EP1	2.70	0.40	0.23			3
EP2.5	2.19	0.19	0.11			3
EP5	2.10	0.16	0.09			3

Fig3E						
Ct	100.00	5.31	2.66	One way ANOVA post-hoc test	**P <0.01 ##P <0.01 VS 6OHDA	4
6OHDA	274.73	34.28	17.14			4
EP1	229.13	31.20	15.60			4
EP2.5	195.21	27.79	13.89			4
EP5	174.45	42.62	21.31			4
Fig4B						
Ct	1.00	0.05	0.03	One way ANOVA post-hoc test	**P <0.01 #P <0.05VS 6OHDA	3
6OHDA	0.51	0.06	0.04			3
EP1	0.64	0.06	0.04			3
EP2.5	0.75	0.07	0.04			3
EP5	0.72	0.06	0.04			3
Fig4C						
Ct	1.00	0.05	0.03	One way ANOVA post-hoc test	##P <0.01 VS 6OHDA	3
6OHDA	1.06	0.17	0.10			3
EP1	1.13	0.15	0.09			3
EP2.5	1.36	0.14	0.08			3
EP5	1.25	0.10	0.06			3
Fig4D						
Ct	1.00	0.05	0.03	One way ANOVA post-hoc test	#P <0.05VS 6OHDA ##P <0.01 VS 6OHDA	3
6OHDA	0.88	0.17	0.10			3
EP1	1.11	0.10	0.06			3
EP2.5	1.22	0.17	0.10			3
EP5	1.24	0.28	0.16			3
