# Supplementary material for: Ethyl pyruvate protects SHSY5Y cells against 6-hydroxydopamine-induced neurotoxicity by upregulating autophagy
Source: PLoS One. 2023 Feb 16;18(2):e0281957. doi: 10.1371/journal.pone.0281957 (PMC9934379; doi:10.1371/journal.pone.0281957)

Fig2 b (Caspase 3)

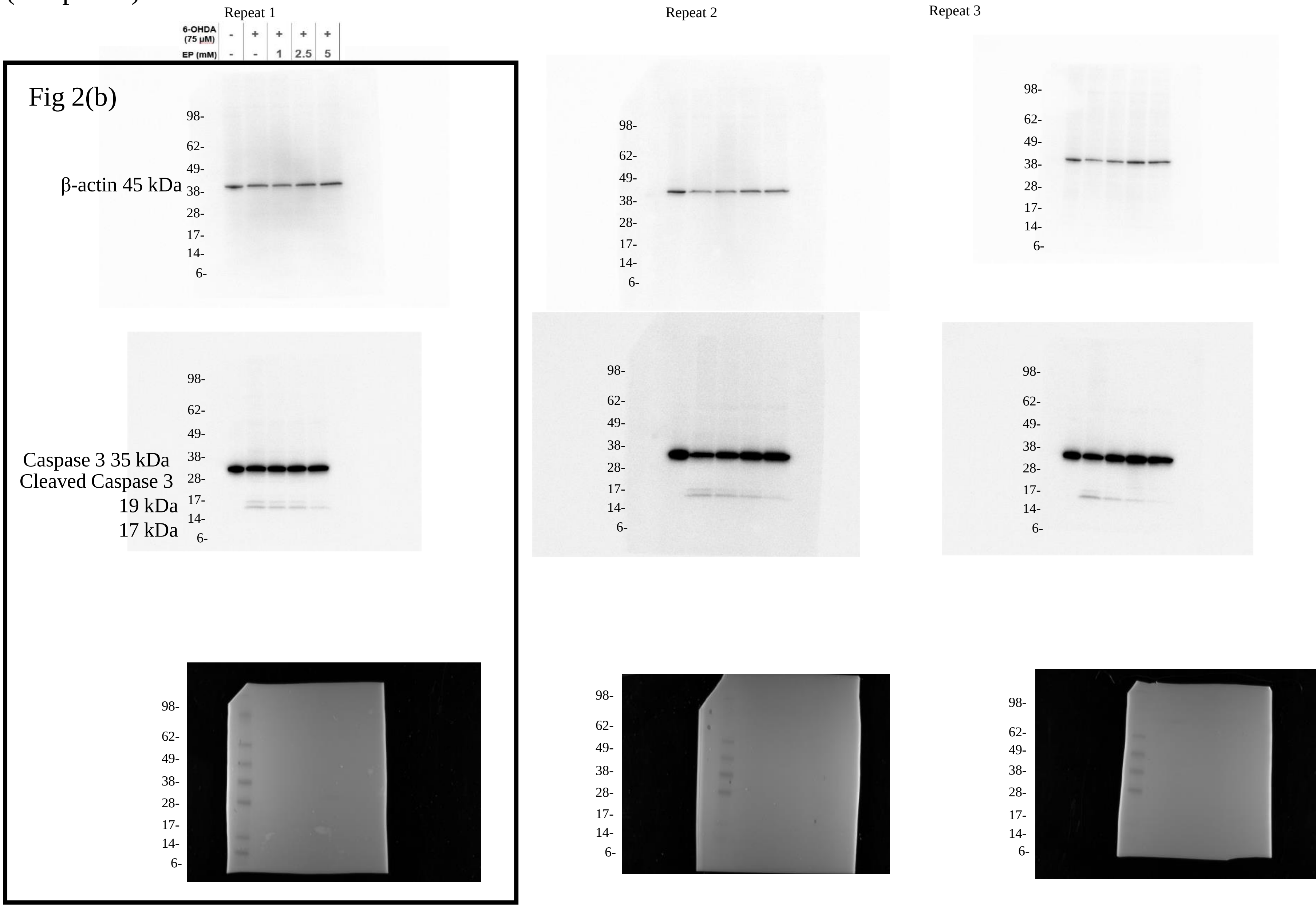

Fig3 b (pERK/ERK)

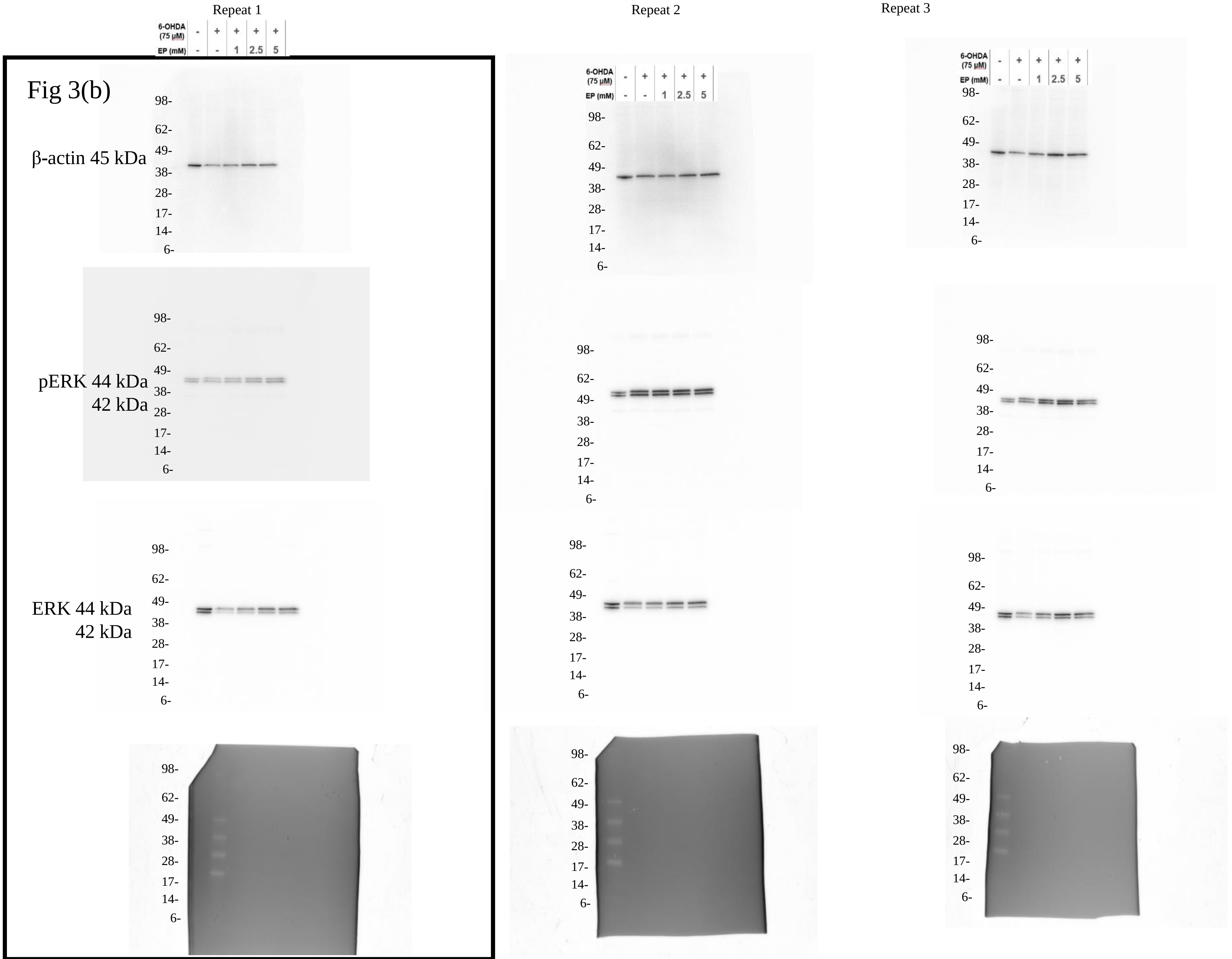

Fig4 a (Beclin1 and LC3)

Repeat 1 Repeat 2 Repeat 3

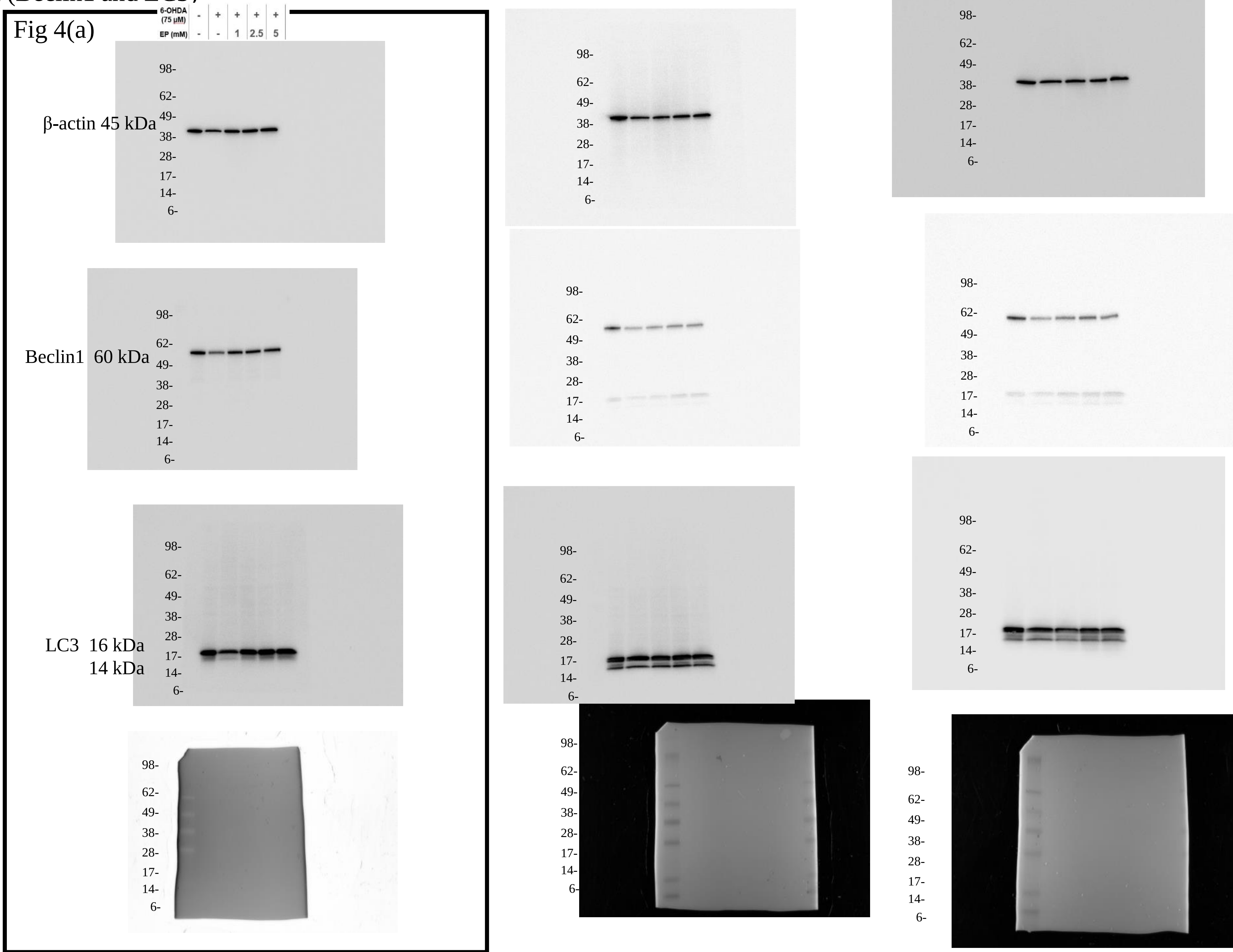

Supplement: S1 Raw data — (ZIP) [file pone.0281957.s001.zip › PLOS_One-S1_raw_images.pdf]
